# Identification of Salt-Tolerant Germplasm and Salt-Responsive Genes in *Brassica napus* Through Phenotypic and Transcriptomic Analyses

**DOI:** 10.3390/antiox15070909

**Published:** 2026-07-22

**Authors:** Lingyu Li, Qi Zhang, Le Tang, Gang Xiao, Lichao Deng, Zhenqian Zhang

**Affiliations:** 1 College of Agronomy, Hunan Agricultural University, Changsha 410128, China; lilingyu199704@163.com (L.L.); XZM100111@163.com (Q.Z.); tangle2219@163.com (L.T.); xiaogang@hunau.edu.cn (G.X.); 2 Hunan Provincial Crop Research Institute, Changsha 410125, China

**Keywords:** *Brassica napus*, salt tolerance, *BnaA02g04730D*, heterologous expression in yeast, overexpression

## Abstract

China’s edible oil self-sufficiency rate is only about 30%, and approximately 100 million acres of saline-alkali land are potentially available for rapeseed cultivation. However, salt stress severely inhibits rapeseed growth and yield, making the breeding of salt-tolerant varieties and the study of underlying molecular mechanisms urgent priorities. In this study, 1609 *Brassica napus* accessions were screened for salt tolerance, and the highly salt-tolerant material ‘Xiangnong Saline-Alkali Oil No. 1’ (XNSA01) was identified. Subsequent physiological, hormonal, transcriptomic, and functional analyses were conducted to characterize its salt-tolerance mechanisms. Salt stress significantly activated antioxidant defense and stress-related hormone responses in XNSA01, accompanied by significant accumulation of proline, abscisic acid, and salicylic acid. Transcriptome analysis identified five candidate salt-responsive genes, among which *BnaA10g15320D*, *BnaA02g04730D*, and *BnaC07g08360D* were closely associated with antioxidant enzyme activities and endogenous hormone contents. In particular, *BnaA02g04730D* showed a significant negative correlation with MDA content. Yeast heterologous expression and transgenic validation further demonstrated that *BnaA02g04730D* enhanced salt tolerance by increasing antioxidant enzyme activities and reducing membrane lipid peroxidation. These findings provide a useful basis for the breeding of salt-tolerant rapeseed varieties and for further investigation of salt-tolerance mechanisms in *B. napus*.

## 1. Introduction

Currently, the self-sufficiency rate of edible oil in China is only approximately 30%, and a severe imbalance exists between supply and demand [1]. Rapeseed (*Brassica napus*) serves as the primary source of domestically produced vegetable oil in China, accounting for nearly 50% of the total oil production from domestic oil-bearing crops [2]. Consequently, increasing the planting area of rapeseed is considered a crucial pathway to enhance oil yield and ensure edible oil security in China. However, arable land resources in China are currently limited. Approximately 6.67 million hectares of saline-alkali land suitable for rapeseed production [3] have emerged as a critical resource for exploiting the potential of rapeseed cultivation expansion [1]. Nevertheless, saline-alkali soils are complex environments and are frequently accompanied by surface salt accumulation. Rapeseed seeds are highly sensitive to salt stress during the germination and seedling stages [4]. Salt stress induces difficulties in water absorption, ion toxicity, and metabolic disorders, directly inhibiting germination potential and seedling establishment, which subsequently affects later growth and development and ultimately leads to significant yield reduction [5,6]. At present, a strategic orientation for the comprehensive utilization of saline-alkali land in China has been established, namely shifting from “remediating saline-alkali land to suit crops” to “breeding salt-tolerant plants to suit saline-alkali land” [7]. Therefore, the screening of elite salt-tolerant germplasm and the breeding of new salt-tolerant rapeseed varieties are of great strategic significance for the effective utilization of saline-alkali land resources, the improvement of rapeseed yield, and the assurance of national edible oil security.

The damage caused by salt stress in plants is mainly manifested as ion toxicity, osmotic stress, and oxidative damage [8]. Under saline conditions, excessive reactive oxygen species (ROS) accumulate in plant cells, resulting in membrane lipid peroxidation and disruption of cell membrane integrity [9]. To alleviate oxidative damage, plants have evolved an antioxidant defense system composed of superoxide dismutase (SOD), peroxidase (POD), catalase (CAT), and other enzymes [10]. Previous studies have shown that salt-tolerant rapeseed varieties can maintain relatively high antioxidant enzyme activities under salt stress, thereby effectively scavenging ROS and reducing malondialdehyde (MDA) accumulation [11,12]. In addition, osmotic adjustment substances such as proline and soluble sugars contribute to the maintenance of cell turgor and normal physiological metabolism under salt stress [11].

Endogenous hormones also play important roles in plant responses to salt stress. Abscisic acid (ABA) is a core stress-responsive hormone, and its accumulation under salt stress can activate SnRK2 protein kinases and regulate downstream stress responses, including stomatal closure and water-loss control [13]. Salicylic acid (SA) has also been reported to participate in stress resistance regulation. Exogenous SA treatment can reduce Na^+^ accumulation and elevate endogenous SA levels in plants, thereby enhancing salt tolerance [14].

Several genes have been confirmed to participate in the regulation of salt tolerance in rapeseed. For example, the overexpression of *OsLTP* or *BADH* significantly improves salt tolerance in rapeseed [15]. Meanwhile, *BnaMPK6* has been verified to play a positive regulatory role in salt tolerance mechanisms [16,17]. In addition, the NHX family and transcription factors such as bZIP, WRKY, and AP2/EREBP have been reported to play important roles in the response to salt stress [18,19]. With the development of high-throughput sequencing technologies, absolute quantitative transcriptome sequencing has been increasingly applied in studies of crop stress resistance, functional gene identification, and molecular breeding improvement because of its high sensitivity and accuracy in transcript quantification [20,21,22]. However, most previous studies on rapeseed salt tolerance have mainly focused on physiological responses or transcriptomic changes alone, whereas relatively few studies have integrated large-scale germplasm screening, physiological traits, and functional validation of key genes.

In addition to the classical salt stress response pathways, increasing evidence suggests that nutrient signaling may also interact with abiotic stress adaptation. Proteins containing the SYG1/Pho81/XPR1 (SPX) domain are recognized as important regulators of phosphate sensing and homeostasis in plants [23]. In *Arabidopsis*, *SPX1* has been identified as an important component of phosphate signaling [23]. Moreover, enhanced expression of *OsSPX1* has been reported to improve stress tolerance in transgenic plants, suggesting that SPX-domain proteins may also participate in broader stress-response networks [24].

Based on the above background, we hypothesized that integrated evaluation of salt tolerance at the phenotypic, physiological, and transcriptomic levels could facilitate the identification of elite salt-tolerant germplasm and candidate salt-responsive genes in *Brassica napus*. Therefore, the objectives of this study were to: (1) screen salt-tolerant germplasm from a large collection of *B. napus* accessions; (2) characterize the physiological responses of the selected material under different salt treatments; and (3) identify and preliminarily validate candidate salt-responsive genes associated with salt tolerance.

## 2. Materials and Methods

### 2.1. Plant Materials

A total of 1609 *Brassica napus* accessions were used in this study (Supplementary Table S1), and all materials were provided by the College of Agronomy, Hunan Agricultural University.

### 2.2. Experimental Methods

#### 2.2.1. Screening of Salt-Tolerant Germplasm

Salt tolerance screening was performed on 1609 rapeseed accessions according to the methods described in the “Technical Regulation for Identification and Evaluation of Salt Tolerance in Rapeseed” (DB32/T 3278-2017) [25]. Salt-tolerant materials were selected based on indices including germination potential, germination rate, and fresh weight [12].

#### 2.2.2. Analysis of Physiological Mechanisms

Pre-germinated seeds of XNSA01 were sown in nutrient soil. Five plants were maintained in each pot, and four pots were prepared for each treatment. Among them, three pots were used as independent biological replicates for physiological and biochemical analyses, while the remaining pot served as a reserve for sampling supplementation if needed. At the 2–3 leaf stage, treatments with varying NaCl concentrations were administered: CK (freshwater control), Y1 (0.12% NaCl), Y2 (0.24% NaCl), and Y3 (0.36% NaCl). The selected NaCl concentrations were determined based on preliminary experiments and were intended to simulate mild-to-moderate salt stress conditions during early growth of rapeseed. Sampling was conducted at 12, 24, 36, and 48 days after treatment. Root and leaf tissues were harvested, flash-frozen in liquid nitrogen, and subsequently stored at −80 °C.

The activities of SOD, POD, and CAT were determined using the nitroblue tetrazolium (NBT) photoreduction method, guaiacol method, and UV absorption method, respectively [12]. MDA content was measured using the thiobarbituric acid (TBA) assay [11]. Soluble protein content was determined using the Coomassie Brilliant Blue G-250 staining method. Ascorbate peroxidase (APX) and glutathione peroxidase (GPX) activities, proline (Pro) content, and endogenous hormone contents (ABA, SA, GA, and IAA) were measured using corresponding enzyme-linked immunosorbent assay (ELISA) kits [26,27].

#### 2.2.3. Identification of Key Salt-Tolerant Genes Based on Transcriptome Analysis

XNSA01 was grown in soil treated with different NaCl concentrations (CK: freshwater control; Y1: 0.12% NaCl; Y2: 0.24% NaCl; Y3: 0.36% NaCl). Root samples were collected after 12 days of treatment. Libraries were constructed using absolute quantitative transcriptomic sequencing technology and subjected to high-throughput sequencing [21]. Bioinformatics analysis was performed to identify differentially expressed genes (DEGs) that responded significantly to salt stress, and these DEGs were further screened as candidate salt-tolerant genes. Total RNA was extracted using the TransZol Up Plus RNA Kit (TransGen Biotech, Beijing, China) and reverse-transcribed into cDNA using the One-Step gDNA Removal and cDNA Synthesis SuperMix kit according to the manufacturer’s instructions. Expression levels of five candidate genes were detected by qRT-PCR (primers listed in Supplementary Table S2), using *BnActin* as the internal reference gene. The stability of *BnActin* under different treatments was evaluated based on the consistency of Ct values across samples before its use as the internal control. Relative gene expression was calculated using the $2^{-\Delta \Delta C_{t}}$ method [28], and correlation analysis was conducted between gene expression levels and physiological indicators.

#### 2.2.4. Validation of Candidate Salt-Tolerant Gene *BnaA02g04730D* and Construction of Transgenic Material

##### Construction of Yeast Expression Vectors and Salt Tolerance Characterization

Total RNA was extracted from roots of XNSA01 and reverse transcribed into cDNA. The candidate gene CDS sequence was amplified and ligated into the pYES2-NTB vector to construct a recombinant yeast expression vector. The recombinant plasmid was transformed into *Saccharomyces cerevisiae* strain INVSc1 using the lithium acetate method [29]. Because yeast cells generally tolerate a wider external salt concentration range than higher plants, a relatively high NaCl concentration was used in the heterologous assay to effectively distinguish salt tolerance differences between transformed and control strains. Growth of the transformed strains on SG-Ura medium containing 1.0 mol· $L^{-1}$ NaCl was compared via spot plate assays to identify the salt tolerance function of the gene.

##### Construction of *BnaA02g04730D* Overexpression Vectors and Genetic Transformation

The coding sequence of *BnaA02g04730D* was inserted into the overexpression vector PC1300s-SPX, in which transgene expression was driven by the CaMV 35S promoter. The recombinant vector was introduced into *Agrobacterium tumefaciens* strain GV3101 and then transformed into hypocotyl explants of *Brassica napus* ‘Zhongshuang 11’ using Agrobacterium-mediated transformation [30].

##### Identification of Transgenic Positive Plants and Expression Level Analysis

Leaf genomic DNA was extracted by a plant genomic DNA extraction kit according to the manufacturer’s instructions, and positive transgenic plants were screened by PCR amplification using specific primers for the hygromycin resistance gene (*HYG*) (Forward: 5’-ACACTACATGGCGTGATTTCAT-3’; Reverse: 5’-TCCACTATCGGCGAGTACTTCT-3’). Total RNA was extracted from the identified positive plants and reverse-transcribed into cDNA. The expression level of the target gene was detected via qRT-PCR. The $2^{-\Delta \Delta C_{t}}$ method [28] was employed to calculate relative gene expression.

#### 2.2.5. Analysis of Salt Tolerance Phenotypes and Physiological Indicators in Genetically Modified Rapeseed

T0-generation overexpressing positive plants and wild-type (WT) plants with consistent growth were selected. During the seedling stage, transgenic lines and wild-type (WT) plants were subjected to 0.24% NaCl treatment, and their growth phenotypes were recorded 12 days later. Relevant physiological and biochemical indicators, including SOD, POD, and CAT activities as well as MDA content, were determined, and all assays were performed as described in Section 2.2.2. The phenotypic and physiological differences between overexpressing plants and wild-type plants under salt stress was compared to evaluate the effect of the *BnaA02g04730D* gene.

#### 2.2.6. Data Statistics and Analysis

All experiments in this study were performed with three biological replicates, and the data are presented as the mean ± standard deviation (SD). Microsoft Excel was used for data collection and preliminary organization. Statistical analyses were conducted using SPSS 27.0 software. Differences among treatments were analyzed by one-way analysis of variance (ANOVA), followed by Duncan’s multiple range test for multiple comparisons. Differences were considered statistically significant at p<0.05 and highly significant at p<0.01. Pearson correlation analysis was performed to evaluate the relationships between gene expression levels and physiological indicators. Gene expression heatmaps were generated using TBtools [31].

## 3. Results and Analysis

Screening of Salt-Tolerant Germplasm

**Figure 1:**
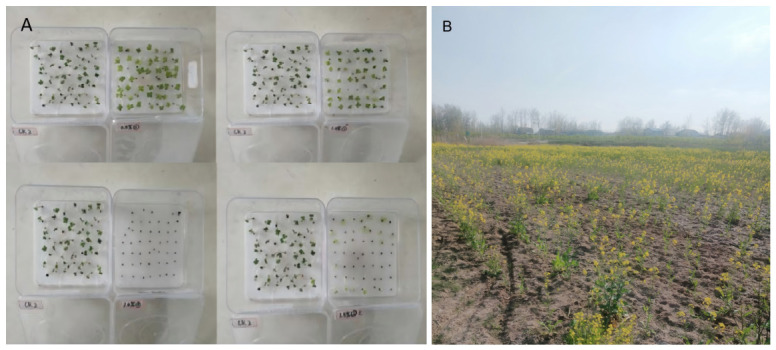
Screening and phenotypic identification of salt-tolerant rapeseed materials. Note: (**A**). Phenotypic differences among some *Brassica napus* materials germinated for 7 days under 1.5% NaCl stress; (**B**). Field growth performance of the selected salt-tolerant accession XNSA01 in saline-alkali soil.

The salt tolerance of 1609 accessions was evaluated during the germination stage using the method described in the Section 2. Based on the overall performance of each accession under salt stress (Figure 1A), 50 accessions were preliminarily screened into high, medium, and low salt-tolerant groups (Supplementary Table S2).

As shown in Figure 1B, the selected accession exhibited favorable growth performance in saline-alkali soil at Sunfuji Township, Liangyuan District, Shangqiu City, Henan Province (34.5920° N, 115.4983° E), where the total soluble salt content of the soil was 52.36 g· kg^−1^. This screening identified the highly salt-tolerant material ‘Xiangnong Saline-Alkali Oil No. 1’, hereafter abbreviated as XNSA01. Subsequent analysis confirmed that XNSA01 is a high-oil (49.33%), double-low material, with 0% erucic acid and 31.79 µmol· g^−1^ glucosinolates. Furthermore, XNSA01 exhibits excellent agronomic traits, including 221.6 pods per plant and a thousand-seed weight of 4.60 g.

### 3.1. Phenotypic and Physiological Responses Under Different NaCl Treatments

#### 3.1.1. Growth Phenotypic Characteristics

**Figure 2:**
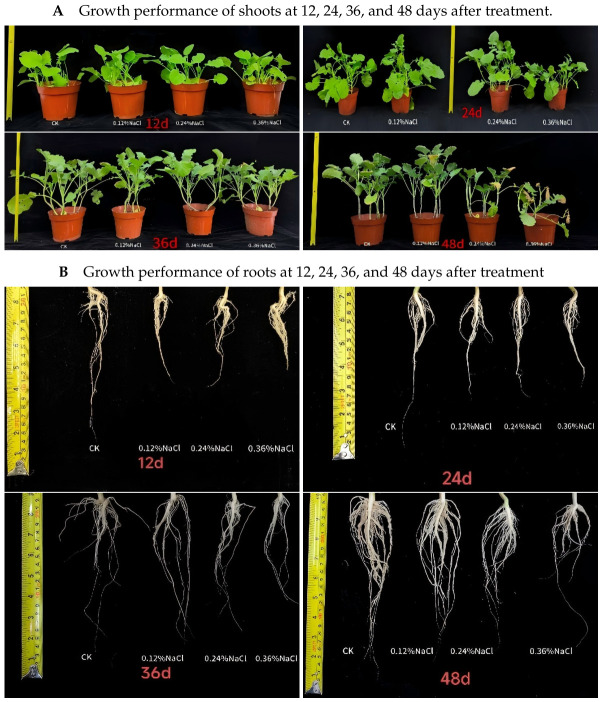
Growth Phenotype of XNSA01 under different NaCl concentrations. Note: From left to right, the treatments are CK (freshwater control), Y1 (0.12% NaCl), Y2 (0.24% NaCl), and Y3 (0.36% NaCl).

Growth phenotypes of XNSA01 under different salt concentrations are shown in Figure 2. Strong adaptability was exhibited by the plants under the Y1 treatment. Plants showed only slight growth inhibition at 12 d. As the adaptation period prolonged, the growth rate gradually recovered. Slight leaf chlorosis was noted only under long-term stress (48 d). However, under Y2 and Y3 treatments, plant growth was significantly inhibited, and the onset of inhibition occurred earlier. In the Y3 treatment group, growth retardation was exhibited as early as 12 d. By 48 d, the whole plant showed severe chlorosis, leaf margin scorching, and even wilting. Primary root development was significantly hindered with increasing salt concentrations. Lateral root density increased during the initial stress stage (12–24 d). This suggests that root system architecture was remodeled to enhance absorption capacity for stress adaptation [27]. However, lateral root growth was also severely inhibited during the late stage of long-term high-salt stress (36–48 d), ultimately resulting in irreversible damage. Under long-term salt stress, partial adaptation may be achieved by plants under low salt concentrations. Conversely, irreversible damage may be induced by high salt concentrations [32].

#### 3.1.2. Differences in Physiological Indicators Under Different Salt Treatments

**Figure 3:**
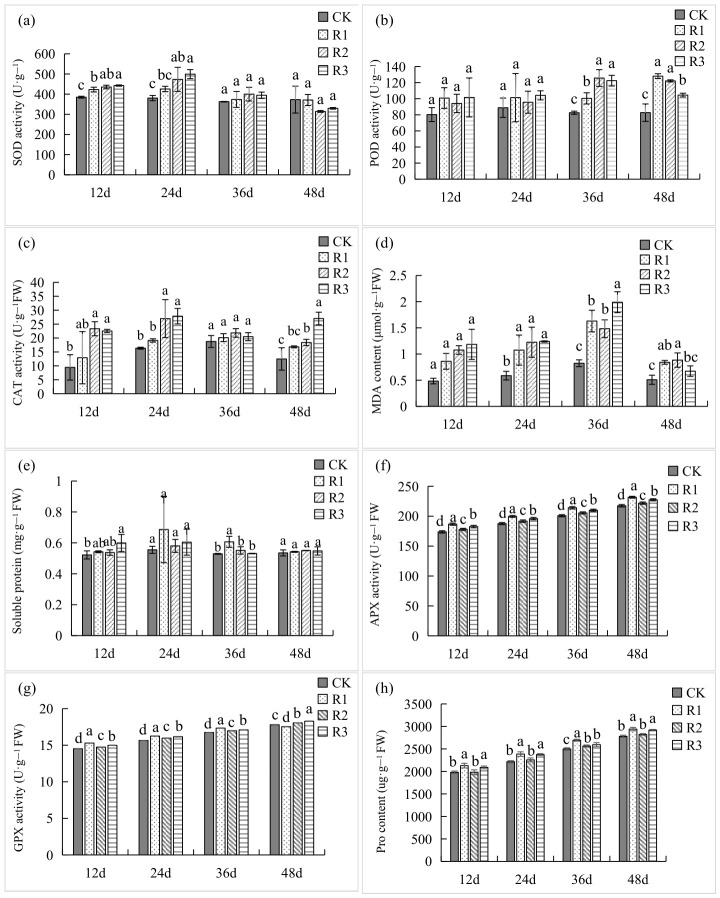
Root physiological parameter content under different salt concentration treatments. Note: (**a**), SOD activity; (**b**), POD activity; (**c**), CAT activity; (**d**), MDA content; (**e**), soluble protein content; (**f**), APX activity; (**g**), GPX activity; (**h**), Pro content. Data are expressed as mean ± SD (n=3). Different lowercase letters indicate significant differences among treatments at p<0.05. CK denotes the freshwater control; R1, R2, and R3 represent root samples treated with Y1 (0.12% NaCl), Y2 (0.24% NaCl), and Y3 (0.36% NaCl), respectively.

**Figure 4:**
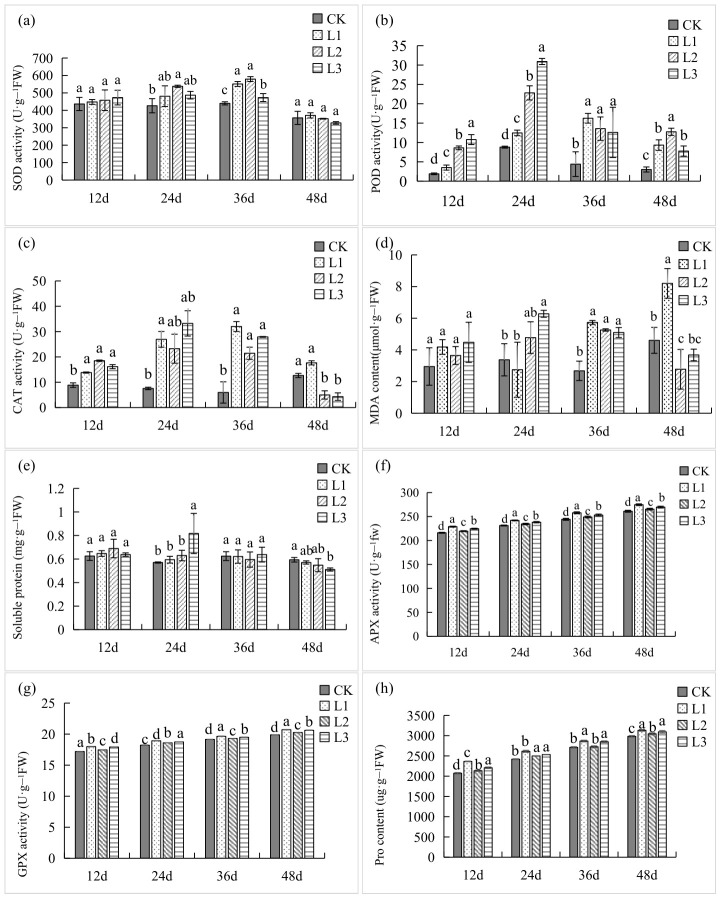
Leaf physiological parameter content under different salt concentration treatments. Note: (**a**), SOD activity; (**b**), POD activity; (**c**), CAT activity; (**d**), MDA content; (**e**), soluble protein content; (**f**), APX activity; (**g**), GPX activity; (**h**), Pro content. Data are expressed as mean ± SD (n=3). Different lowercase letters indicate significant differences among treatments at p<0.05. CK denotes the freshwater control; L1, L2, and L3 represent leaf samples treated with Y1 (0.12% NaCl), Y2 (0.24% NaCl), and Y3 (0.36% NaCl), respectively.

Root and leaf physiological parameter contents under different salt concentration treatments are shown in Figure 3 and Figure 4, respectively.

**Figure 5:**
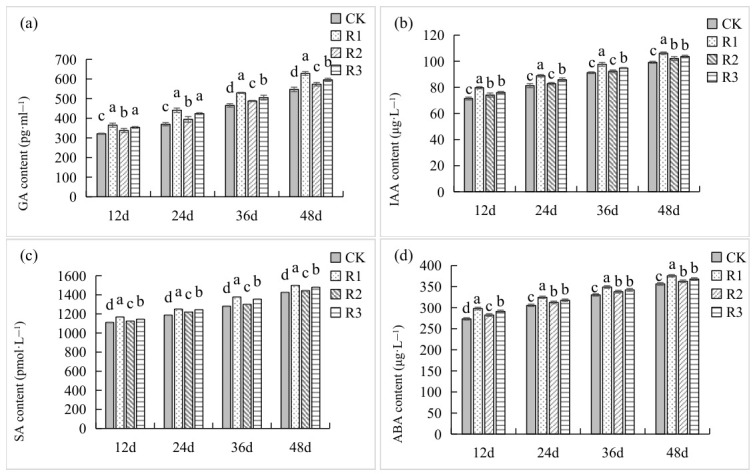
Endogenous hormone contents in roots under different NaCl concentrations. Note: (**a**), GA content; (**b**), IAA content; (**c**), SA content; (**d**), ABA content. Data are expressed as mean ± SD (n=3). Different lowercase letters indicate significant differences among treatments at p<0.05. R1, R2, and R3 represent root samples treated with Y1 (0.12% NaCl), Y2 (0.24% NaCl), and Y3 (0.36% NaCl), respectively.

As shown in Figure 5, the concentrations of all four hormones exhibited an overall upward trend with increasing salt concentration and treatment duration. Among the four hormones, abscisic acid (ABA), a core signaling molecule in plant responses to abiotic stress, showed an overall increase under salt treatment, particularly at the later stage (48 d). Compared with the control, ABA levels remained relatively high in all salt-treated groups, indicating that salt stress induced ABA accumulation to help plants cope with adverse conditions [33]. This response may contribute to stomatal regulation and the activation of stress-responsive genes [13]. Salicylic acid (SA) content was significantly higher in the Y2 and Y3 treatment groups than in the control, and continued to accumulate over time. At 48 days, SA content reached 1498.58 pmol· $L^{-1}$ in the Y1 treatment group, 1442.99 pmol· $L^{-1}$ in the Y2 treatment group, and 1478.68 pmol· $L^{-1}$ in the Y3 treatment group, all of which were higher than the CK value (1424.31 pmol· $L^{-1}$). These results indicate that salt stress stimulated SA accumulation, especially under salt-treated conditions, thereby enhancing stress resistance [27]. Although auxin (IAA) and gibberellin (GA) levels generally increased, their growth slowed or even slightly decreased in the later stages of Y3 treatment. At 48 days, GA content in the Y1 treatment group reached 628 pg· ${\mathrm{mL}}^{-1}$, which was 14.8% higher than that of CK (547 pg· ${\mathrm{mL}}^{-1}$) at the same time point. The Y3 treatment group recorded 596 pg· mL^−1^, which was higher than CK but significantly lower than Y1. Auxin (IAA) peaked at 106 µg· $L^{-1}$ in the Y1 treatment group at 48 days, markedly exceeding CK (99 µg· $L^{-1}$) and both Y2 and Y3 groups. This suggests that low-concentration salt stress may promote root growth to adapt to adverse conditions by maintaining higher levels of GA and IAA, whereas higher salt concentrations may exert a certain inhibitory effect on plants [13].

### 3.2. Screening of Key Salt-Tolerant Genes Based on Quantitative Transcriptome Sequencing

#### 3.2.1. Screening of Key Salt-Tolerant Genes Based on Transcriptome Sequencing

Transcriptome sequencing with absolute quantification was performed on root samples of XNSA01 after 12 days of treatment under different salt concentrations. A total of 2380, 2737, and 8715 differentially expressed genes (DEGs) were identified across the three treatments, respectively. Among these, 713 core differentially expressed genes (DEGs) were identified across the comparisons, including 483 upregulated genes and 230 downregulated genes.

Previous research has shown that plants activate a series of complex metabolic pathways under salt stress to maintain cellular homeostasis [34]. For instance, the phenylpropanoid biosynthesis pathway has been demonstrated to enhance plant salt tolerance by synthesizing secondary metabolites such as lignin and flavonoids, thereby increasing cell wall rigidity and scavenging reactive oxygen species [35]; plant hormone signal transduction, particularly the abscisic acid (ABA) and ethylene signaling pathways, plays a crucial role in regulating stomatal closure and stress gene expression [36]; the MAPK signaling pathway also serves as a key hub in plant responses to salt stress, participating in the regulation of downstream stress-resistant gene expression [37]. Based on the above research background, functional enrichment analysis was performed on the differentially expressed genes, and the enrichment results are presented in Supplementary Figure S1. Results indicate that the identified DEGs in this study were significantly enriched in pathways such as ‘phenylpropanoid biosynthesis’, ‘plant hormone signal transduction’ and ‘metabolic pathways’. Notably, under Y3 treatment, substantial gene expression changes occurred in pathways related to oxidoreductase activity and transmembrane transporter activity. These findings indicate that XNSA01 primarily adapts to saline stress through multiple mechanisms, including activation of phenylpropanoid metabolism, hormone signaling, and regulation of ion transport. Therefore, candidate genes responding to salt stress were prioritized from these significantly enriched key pathways.

A preliminary screening of expression levels was conducted on 713 core genes, using the criteria of absolute logFC > 1.5 and *p*-value < 0.05 to focus on genes exhibiting the most intense responses to salt stress. Subsequently, functional annotation screening was performed based on GO and KEGG enrichment analysis results, prioritizing genes closely associated with plant salt tolerance mechanisms. The specific screening criteria included: (1) genes annotated as transcription factors (e.g., AP2/ERF, MYB, WRKY), which may initiate the entire salt tolerance response as upstream regulators; (2) genes involved in antioxidant systems (e.g., peroxidase, glutathione S-transferase); (3) genes participating in ion transport and osmotic adjustment (e.g., SPX domain-containing proteins); and (4) genes involved in hormone signal transduction (e.g., auxin-responsive proteins). Twenty differential genes were identified by combining gene annotations and NCBI database screening (Supplementary Table S4).

Under salt stress, the excessive uptake of ${\mathrm{Na}}^{+}$ competitively inhibits the absorption of other nutrient ions, rendering plants unable to grow normally [8]. Previous studies have investigated ion osmotic adjustment, antioxidant enzyme regulation, and the impact on phosphate transporters under salt stress [38]. Based on these findings, genes with functional annotations related to salt tolerance were screened from the differentially expressed genes. Further screening was conducted by integrating gene function information from the NCBI database. Consequently, five key differential genes were identified: *BnaA07g33310D*, *BnaA10g15320D*, *BnaA02g04730D*, *BnaC03g23980D*, and *BnaC07g08360D*. Among the five candidate genes, *BnaA10g15320D*, *BnaA02g04730D*, and *BnaC07g08360D* were selected for subsequent functional validation based on their combined expression responsiveness under salt stress, correlation with physiological indicators, functional annotation, and experimental feasibility. The remaining two genes, including *BnaC03g23980D* and *BnaA07g33310D*, also showed potential relevance to salt stress and warrant further investigation in future studies.

#### 3.2.2. Expression Patterns of Candidate Key Genes Under Different Salt Treatments

**Figure 6:**
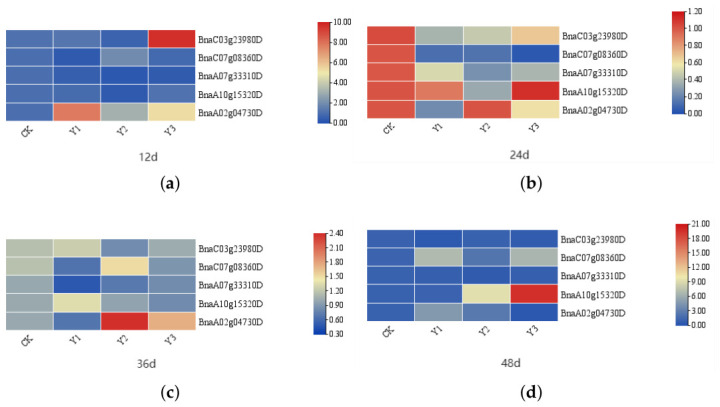
Expression patterns of five key salt-responsive genes under different NaCl treatments. Note: (**a**) 12 days of treatment; (**b**) 24 days of treatment; (**c**) 36 days of treatment; (**d**) 48 days of treatment. The color scale in each panel was adjusted independently to better visualize expression variation within each time point.

The root system is a key site for salt stress response [39]. Expression patterns of the five candidate genes under different salt concentrations were analyzed by qRT-PCR (Figure 6).

As shown in Figure 6, all five genes were significantly induced by salt stress, though their response patterns differed. Specifically, *BnaA02g04730D* exhibited suppressed expression during the early stages of Y1 treatment. Under Y2 and Y3 treatments, although initially downregulated, its expression showed an adaptive recovery trend over time. In contrast, *BnaA10g15320D* exhibited a sharp increase in expression after 48 days of treatment. Similarly, *BnaC03g23980D* reached 19.67-fold expression relative to the control at 48 days under Y3 treatment. These results confirm that the selected candidate genes indeed participate in the molecular response of rapeseed to salt stress. *BnaC07g08360D* showed significant upregulation only during the early stage of Y1 treatment, exhibiting substantial fluctuations under other conditions, suggesting this gene may be associated with specific responses. *BnaA10g15320D* exhibited extreme expression variations in both Y1 and Y3 treatments, suggesting potential regulatory roles in acute stress responses. Based on these expression patterns, genes like *BnaA02g04730D* are hypothesized to occupy central positions in the rapeseed salt tolerance regulatory network.

#### 3.2.3. Correlation Analysis of Key Gene Expression Levels with Physiological Indicators and Hormone Content

Correlation analysis was performed between the expression levels of five key genes and root physiological indicators as well as endogenous hormone content (Table 1).

As shown in Table 1, the expression level of *BnaA02g04730D* showed significant or highly significant negative correlations with SOD activity, CAT activity, and MDA content at the early stages of treatment (12 d and 24 d), suggesting a close association of this gene with antioxidant regulation and membrane lipid peroxidation under salt stress. In addition, the expression of *BnaA02g04730D* showed highly significant negative correlations with ABA and GA contents at 36 d (Table 2), indicating that this gene may also be involved in hormone-related regulatory pathways. The expression of gene *BnaA10g15320D* showed a significant negative correlation with multiple antioxidant indicators at 24 days of treatment, but turned into a highly significant positive correlation with APX activity and Pro content at 48 days (r > 0.9). The expression of gene *BnaC07g08360D* showed a very significant positive correlation with GPX, APX activity and Pro content on the 12th day of treatment, and a very significant negative correlation with GPX, APX activity and Pro content on the 24th day. In the early stage, it showed a very significant positive correlation with the content of hormones (IAA, ABA, etc.), but the correlation turned to a negative correlation as time went by.

Therefore, the significant expression changes and strong correlations with physiological and biochemical indicators observed in the *BnaA02g04730D*, *BnaA10g15320D*, and *BnaC07g08360D* genes suggest that these may be key genes influencing rapeseed salt tolerance.

### 3.3. Identification and Functional Validation of Key Salt-Tolerance Genes

#### 3.3.1. Yeast Phenotyping of Key Salt Tolerance Genes

**Figure 7:**
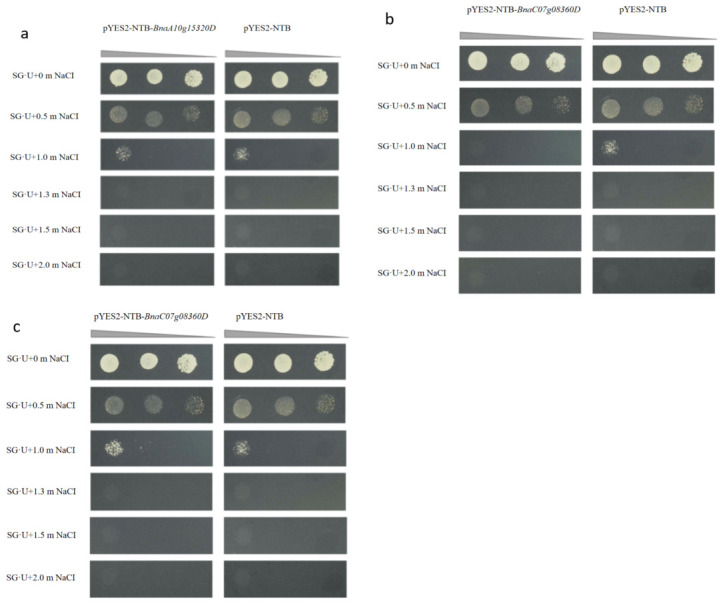
Functional validation of candidate key genes for salt tolerance in yeast. Note: Yeast cultures transformed with recombinant plasmids (pYES2-NTB-target gene) and the empty vector control (pYES2-NTB) were serially diluted (100, $10^{-1}$, $10^{-2}$) and spotted onto SG-U medium containing different concentrations of NaCl (0–2.0 mol· $L^{-1}$). (**a**): *BnaC07g08360D*; (**b**): *BnaA10g15320D*; (**c**): *BnaA02g04730D*.

Yeast expression vectors (pYES2-NTB) carrying *BnaA10g15320D*, *BnaA02g04730D*, and *BnaC07g08360D* were constructed and transformed into the *Saccharomyces cerevisiae* INVSc1 strain (Figure 7).

As shown in Figure 7, all strains exhibited uniform growth phenotypes on the normal medium (SC-U). In contrast, significant growth differences were exhibited on the stress medium containing 1.0 mol· $L^{-1}$ NaCl. The strongest growth vigor was displayed by the strain expressing *BnaA02g04730D*, which was significantly superior to the empty vector control (*pYES2-NTB*). This indicates that the salt tolerance of yeast cells is substantially enhanced by this gene. Conversely, growth inhibition was observed in the strain expressing *BnaA10g15320D*, where no significant difference was found compared to the control. Furthermore, the growth vigor of the strain expressing *BnaC07g08360D* was even weaker than that of the control. Consequently, *BnaA02g04730D* was identified as a key positive regulator of salt tolerance in *Brassica napus*.

#### 3.3.2. Identification and Expression Analysis of Transgenic Plants

Using agrobacterium-mediated transformation of *Brassica napus* ‘Zhongshuang 11’, *BnaA02g04730D*-overexpressing regenerated plants were obtained. Genomic DNA extracted from regenerated plant leaves was subjected to PCR using specific primers targeting the hygromycin resistance gene (*HYG*). The results showed that a specific band of approximately 557 bp was amplified using the recombinant plasmid as a positive control, while no amplification bands were detected in negative controls (wild-type plants and water). Among the 16 regenerated plants tested, 14 amplified a target band identical to the positive control, yielding a positive rate of 87.5%. These results confirm the successful integration of the exogenous gene into the genomes of these transgenic plants.

**Figure 8:**
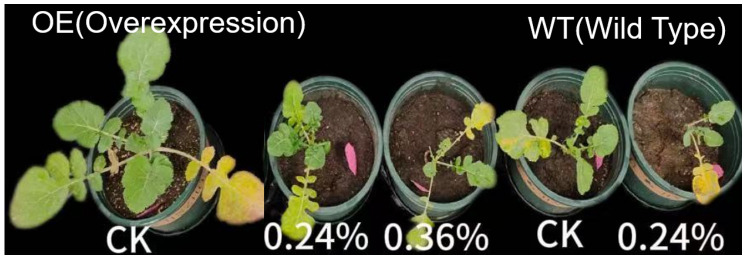
Representative phenotypes of *BnaA02g04730D* overexpression lines under salt stress.

As shown in Figure 8, under control conditions (CK), the OE lines displayed normal growth comparable to that of the WT plants, indicating that overexpression of *BnaA02g04730D* did not cause obvious growth penalties under non-stress conditions. After 12 d of Y2 treatment, WT plants exhibited visible growth inhibition, including stunted growth and leaf chlorosis. In contrast, the OE plants showed relatively milder stress symptoms and maintained better overall growth status under the same treatment. These phenotypic observations suggest that overexpression of *BnaA02g04730D* may alleviate salt-stress damage in rapeseed.

Three PCR-positive lines (OE #12, #4, #5) and the wild-type (WT) were selected for qRT-PCR analysis.

**Figure 9:**
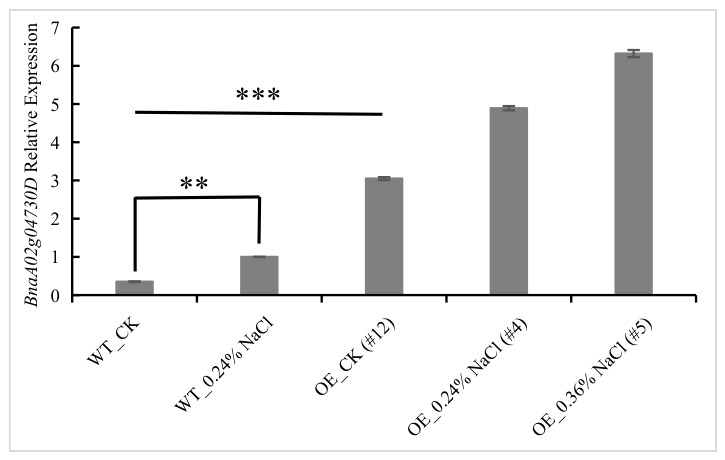
Relative expression level of *BnaA02g04730D* in wild-type (WT) and overexpression (OE) lines. Note: ** and *** indicate significant differences at p<0.01 and p<0.001, respectively.

As shown in Figure 9, the relative expression levels of *BnaA02g04730D* in the three transgenic lines under normal growth conditions (CK) were significantly higher than those in the wild type (WT). The highest expression was observed in the OE #12 line, which was approximately 8.7-fold higher than that of the WT. This confirms that effective constitutive high-level expression of the gene was achieved in the transgenic plants. Furthermore, in the WT, gene expression was upregulated by approximately 2.9-fold under the Y2 treatment, indicating that *BnaA02g04730D* is a salt stress-inducible gene. Higher transcript abundance of *BnaA02g04730D* was observed in the tested overexpression lines than in the WT under both control and salt treatment conditions. However, because independent transgenic lines were used in different treatment groups, caution is required when interpreting treatment-dependent differences in expression intensity among these lines. Under the Y2 treatment, expression levels in the OE #4 and OE #5 lines were higher than those under CK conditions and substantially exceeded the levels in the WT under salt stress. Integrating the PCR and qRT-PCR results, transgenic materials with high *BnaA02g04730D* expression were successfully obtained for subsequent functional analysis of salt tolerance.

#### 3.3.3. Analysis of Physiological and Biochemical Differences Under Different Salt Concentrations

**Figure 10:**
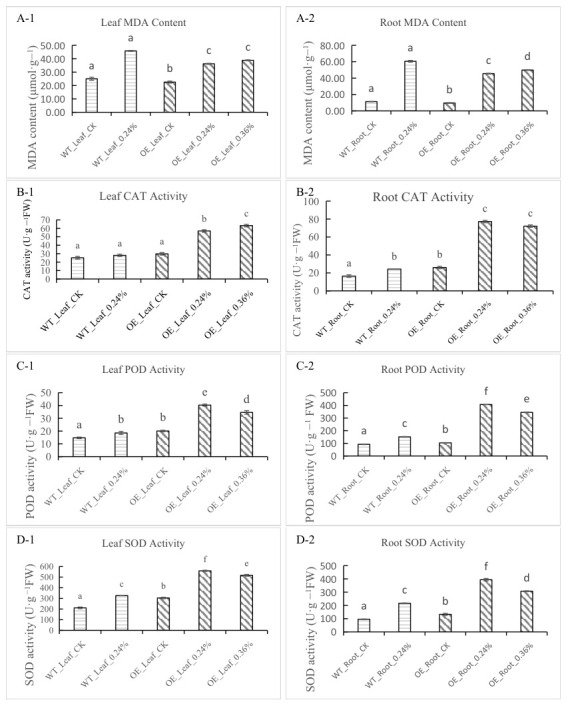
Physiological and biochemical differences between wild-type (WT) and *BnaA02g04730D*-overexpressing (OE) plants under salt stress. Note: (**A-1**), leaf MDA content; (**A-2**), root MDA content; (**B-1**), leaf CAT activity; (**B-2**), root CAT activity; (**C-1**), leaf POD activity; (**C-2**), root POD activity; (**D-1**), leaf SOD activity; (**D-2**), root SOD activity. Data are presented as mean ± SD (n=3). Different lowercase letters indicate significant differences among treatments at p<0.05.

Physiological and biochemical parameters in roots and leaves of WT and OE plants under salt stress were analyzed, and the results are presented in Figure 10.

As shown in Figure 10(A-1,A-2), MDA levels do not differ significantly between WT and OE plants under CK conditions. After Y2 treatment, MDA content increases sharply in WT roots and leaves, whereas its accumulation is significantly suppressed in OE plants. In contrast, MDA accumulation in OE plants was significantly suppressed. Particularly in roots, MDA content in OE plants was significantly lower than in WT (*p* < 0.05), indicating that *BnaA02g04730D* overexpression effectively maintained cell membrane integrity.

Further analysis of the antioxidant enzyme system revealed that *BnaA02g04730D* significantly upregulated SOD, CAT, and POD activities (Figure 10(B-1–D-2)). Under salt stress, OE plants exhibited a more rapid and robust antioxidant response than WT. Under Y2 treatment, the SOD, CAT, and POD activities in the leaves increased to approximately 1.7-fold, 2.0-fold, and 2.1-fold those of the wild-type (WT), respectively; in the roots, the corresponding enzyme activities reached 1.8-fold, 3.1-fold, and 2.7-fold those of the WT, respectively. Even under the higher salt concentration Y3 treatment, OE plants maintained exceptionally high enzyme activity levels. Thus, *BnaA02g04730D* enhances the synergistic activity of SOD, CAT, and POD to establish an efficient reactive oxygen species (ROS) scavenging network. This significantly mitigates oxidative damage to the cell membrane system caused by salt stress, ultimately conferring enhanced salt tolerance to *Brassica napus*.

## 4. Discussion

### 4.1. Screening of Superior Salt-Tolerant Breeding Materials

The selected salt-tolerant accession XNSA01 exhibited superior growth vigor under salt stress. Physiological analysis indicated that the core mechanism of its salt tolerance lies in its efficient and persistent reactive oxygen species (ROS) scavenging capability. Homeostasis under salt stress is maintained through the synergistic regulation of an efficient antioxidant enzyme system and hormones. Field results demonstrated that even under severe saline-alkali conditions (total soluble salt 52.36 g· ${\mathrm{kg}}^{-1}$), the growth period was successfully completed, and robust growth was maintained by this variety, exhibiting favorable agronomic traits. XNSA01 is a high-oil (49.33%), double-low (0% erucic acid and 31.79 µmol· $g^{-1}$ glucosinolates) variety with yellow seeds, which also boasts excellent yield components (221.6 pods per plant). This elite germplasm can facilitate the breeding of new salt-tolerant rapeseed varieties. It should also be noted that evaluation at the germination stage has certain limitations, because early seedling performance is influenced not only by intrinsic salt tolerance but also by seed reserves. Therefore, traits such as germination rate and fresh weight at this stage may not fully reflect the physiological salt tolerance of the genotype. In the present study, this limitation was addressed by combining germination-stage screening with field validation, physiological analyses, transcriptome profiling, and functional verification, thereby improving the reliability of the selected salt-tolerant material.

### 4.2. Molecular Mechanisms of Salt Tolerance in Rapeseed

Salt stress triggered rapid antioxidant responses in both roots and leaves of *Brassica napus*, indicating that ROS scavenging is an important component of salt tolerance in XNSA01. Under prolonged stress, roots appeared to maintain stronger tolerance than leaves, which was associated with sustained antioxidant enzyme activity and osmotic adjustment. In particular, proline accumulation reached 2918 µg· $g^{-1}$ FW under the Y3 treatment, suggesting an important role of osmotic regulation in alleviating salt- induced damage.

Hormonal responses also differed under different salt stress levels. Low-salinity stress was associated with relatively higher levels of growth-related hormones such as GA and IAA, suggesting that mild salt stress may help maintain root growth adaptation. In contrast, high-salinity stress promoted the accumulation of stress-related hormones such as SA and ABA, indicating activation of systemic stress defense pathways.

Through transcriptome screening, yeast assays, and transgenic verification, *BnaA02g04730D*, which contains an SPX domain, was identified as a key salt-tolerance-related gene in rapeseed. SPX-domain proteins are mainly known for their roles in phosphate signaling [23], but increasing evidence suggests that they may also participate in broader abiotic stress responses [24]. In the present study, *BnaA02g04730D* was strongly induced by salt stress and improved salt tolerance in overexpressing plants. Physiological analyses further showed that overexpression of *BnaA02g04730D* enhanced antioxidant enzyme activities and reduced MDA accumulation, indicating that this gene may contribute to salt tolerance by strengthening antioxidant defense and maintaining membrane stability. These findings are consistent with previous studies showing that efficient antioxidant systems are critical for cellular homeostasis under salt stress [11,40].

It should also be noted that the transgenic evaluation in this study was conducted using T0 plants, and independent overexpression lines were used in some expression comparisons. Therefore, the potential influence of line-to-line variation cannot be completely excluded. Further validation using stable homozygous lines in later generations will be necessary. In addition, the phenotypic comparison of transgenic plants in this study was mainly based on representative images. Further quantitative evaluation using growth-related traits such as plant height and biomass would strengthen the phenotypic evidence for salt tolerance.

### 4.3. Integrated Analysis for Screening Salt-Tolerant Rapeseed Materials

Based on transcriptome analysis, a comprehensive screening strategy was established by integrating plant phenotypes, physiological indicators, and gene expression patterns. Particular attention was given to genes showing strong expression changes under salt stress and close associations with antioxidant enzyme activities and membrane damage indicators. The analysis revealed that *BnaA02g04730D* exhibited strong correlations with SOD, CAT, and MDA at multiple time points, suggesting its important role in antioxidant regulation and membrane stability under salt stress. Therefore, integrating transcriptomic data with physiological and biochemical indicators represents an effective approach for identifying candidate salt-tolerance genes. In this study, *BnaA02g04730D* was further validated as a positive regulator of salt tolerance in rapeseed. These results provide a useful basis for the early identification of salt-tolerant germplasm and for the efficient screening of salt-tolerance-related genes in *Brassica napus*.

## 5. Conclusions

A salt tolerance screening of 1609 *Brassica napus* accessions was conducted during the germination stage using a 1.5% NaCl solution. Combined with field validation in saline-alkali soil, the highly salt-tolerant accession XNSA01 was identified. During the early stage of salt stress (24 d), both roots and leaves rapidly activated antioxidant defenses, with significant increases in SOD, CAT, and POD activities reaching peak levels. In the late stage of prolonged stress (48 d), roots exhibited greater tolerance than leaves by maintaining high antioxidant enzyme activities and continuously accumulating proline, while MDA content in roots was effectively controlled. Under low salinity stress, plants primarily promoted root growth adaptation by maintaining elevated levels of growth hormones GA and IAA. In contrast, high salinity stress significantly induced the accumulation of stress hormones SA and ABA, with ABA sustaining high levels during the late stress phase to initiate systemic defense responses. Absolute quantitative transcriptomic analysis revealed that the expression levels of the identified key genes were closely correlated with physiological and biochemical indicators: the expression levels of three candidate genes showed significant correlations (|r| > 0.70) with antioxidant enzyme activities (SOD, CAT, POD) and endogenous hormone contents (ABA, SA). Among these, the key gene *BnaA02g04730D* exhibited a highly significant negative correlation with MDA content (r<−0.81), identifying it as a critical candidate gene responding to salt stress. Both yeast assays and transgenic rapeseed validation confirmed that *BnaA02g04730D* positively regulates salt tolerance in rapeseed. Under the Y2 treatment, SOD, CAT, and POD activities in overexpressing plants were 1.8-, 3.1-, and 2.7-fold higher, respectively, than in the wild type, while MDA content was significantly reduced by approximately 25%. This study identified the salt-tolerant *Brassica napus* cultivar XNSA01 (‘Xiangnong Saline-Alkali Oil No. 1’) and uncovered the key salt-tolerance gene *BnaA02g04730D*. These findings provide a useful basis and valuable genetic resources for screening salt-tolerant germplasm and for further investigating the molecular mechanisms of salt tolerance in rapeseed.

## Figures and Tables

**Table 1:** Correlation Analysis Between Expression Levels of Key Genes and Root Physiological Indicators.

Period	Physiological Indicators			Gene		
		*BnaA07g33310D*	*BnaA10g15320D*	*BnaA02g04730D*	*BnaC03g23980D*	*BnaC07g08360D*
12d	SOD	0.507	0.208	−0.914 **	−0.251	0.544
	POD	0.050	−0.247	−0.400	−0.012	0.513
	CAT	0.404	0.212	−0.703 *	−0.362	0.100
	MDA	0.509	0.130	−0.812 **	−0.199	0.427
	Soluble protein	0.584 *	−0.008	−0.308	0.262	0.314
	GPX	0.364	−0.421	−0.468	0.177	0.992 **
	APX	0.353	−0.289	−0.513	0.164	0.952 **
	Pro	0.524	−0.682 *	−0.245	0.449	0.830 **
24d	SOD	−0.332	−0.715 **	−0.778 **	−0.142	−0.095
	POD	−0.233	−0.294	−0.257	0.155	−0.303
	CAT	−0.355	−0.650 *	−0.749 **	−0.281	0.117
	MDA	−0.565	−0.810 **	−0.832 **	−0.250	−0.254
	Soluble protein	−0.321	−0.263	−0.110	0.018	−0.479
	GPX	−0.716 **	−0.895 **	−0.706 *	0.044	−0.794 **
	APX	−0.631 *	−0.747 **	−0.537	0.173	−0.832 **
	Pro	−0.457	−0.720 **	−0.451	0.334	−0.773 **
36d	SOD	−0.313	0.106	−0.093	−0.327	0.502
	POD	−0.263	0.227	−0.277	−0.293	0.746 **
	CAT	−0.120	0.075	−0.369	−0.142	0.511
	MDA	−0.089	−0.190	−0.474	−0.201	0.262
	Soluble protein	0.234	−0.407	−0.755 **	0.755 **	−0.439
	GPX	0.149	−0.453	−0.848 **	0.452	−0.251
	APX	0.200	−0.391	−0.733 **	0.356	−0.276
	Pro	0.069	−0.395	−0.783 **	0.475	−0.307
48d	SOD	−0.434	−0.066	0.392	−0.486	0.206
	POD	−0.422	0.473	−0.590 *	0.032	0.755 **
	CAT	−0.168	0.573	−0.081	0.880 **	−0.324
	MDA	−0.215	0.280	−0.602 *	0.065	0.655 *
	Soluble protein	−0.149	0.083	−0.261	0.365	0.017
	GPX	0.289	−0.012	−0.259	0.912 **	−0.760 **
	APX	−0.726 **	0.902 **	0.035	0.160	0.519
	Pro	−0.746 **	0.964 **	0.103	0.288	0.379

Note: * and ** indicate significant correlations at p<0.05 and p<0.01, respectively (two-tailed).

**Table 2:** Correlation analysis of key gene expression and root endogenous hormone content.

Period	Indicator			Gene		
		*BnaA07g33310D*	*BnaA10g15320D*	*BnaA02g04730D*	*BnaC03g23980D*	*BnaC07g08360D*
12d	IAA	0.315	−0.268	−0.461	0.160	0.947 **
	SA	0.364	−0.421	−0.425	0.205	0.990 **
	ABA	0.429	−0.297	−0.557	0.167	0.959 **
	GA	0.450	−0.327	−0.546	0.121	0.914 **
24d	IAA	−0.622 *	−0.638 *	−0.377	0.164	−0.958 **
	SA	−0.661 *	−0.897 **	−0.700 *	0.092	−0.796 **
	ABA	−0.667 *	−0.733 **	−0.489	0.078	−0.827 **
	GA	−0.635 *	−0.755 **	−0.522	0.180	−0.856 **
36d	IAA	0.230	−0.451	−0.704 *	0.443	−0.437
	SA	0.189	−0.527	−0.676 *	0.344	−0.341
	ABA	0.236	−0.473	−0.833 **	0.430	−0.227
	GA	0.137	−0.479	−0.820 **	0.362	−0.255
48d	IAA	−0.645 *	0.883 **	−0.148	0.104	0.612 *
	SA	−0.793 **	0.943 **	0.079	0.198	0.510
	ABA	−0.780 **	0.885 **	−0.082	0.043	0.622 *
	GA	−0.649 *	0.891 **	0.030	0.100	0.605 *

Note: * and ** indicate significant correlations at p<0.05 and p<0.01, respectively (two-tailed).

## Data Availability

The materials of this study were provided by the College of Agronomy, Hunan Agricultural University. Correspondence and requests for materials should be addressed to Zhenqian Zhang (zhangzhenqian@hunau.edu.cn).

## Supplementary Materials

The following supporting information can be downloaded at: https://www.mdpi.com/article/10.3390/antiox15070909/s1, Figure S1: GO classification of differentially expressed genes under Y1, Y2, and Y3 salt treatments; Table S1: Germination potential, germination percentage, and fresh weight of 1609 *Brassica napus* accessions under salt stress; Table S2: Primer sequences of candidate differentially expressed genes used for qRT-PCR analysis; Table S3: Salt-tolerant materials screened at different tolerance levels; Table S4: Differentially expressed genes (DEGs) in response to salt stress.

## References

[1] Wang G., Ni G., Feng G., Burril H.M., Li J., Zhang J., Zhang F. (2024). Saline-alkali soil reclamation and utilization in China: Progress and prospects. Front. Agric. Sci. Eng..

[2] Hu Q., Hua W., Yin Y., Zhang X., Liu L., Shi J., Zhao Y., Qin L., Chen C., Wang H. (2017). Rapeseed research and production in China. Crop J..

[3] Yang J. (2008). Development and prospect of the research on salt-affected soils in China. Acta Pedol. Sin..

[4] Chen M., Ren J., Jiang L., Lao P., Yang J., Wang J., Wang L., Zhang Z. (2024). Comprehensive evaluation of salt tolerance during germination in cabbage-type rapeseed varieties. Chin. J. Oil Crop. Sci..

[5] Zhu J.K. (2001). Plant salt tolerance. Trends Plant Sci..

[6] Wang W., Zhang F., Sun L., Yang L., Yang Y., Wang Y., Siddique K.H., Pang J. (2022). Alkaline salt inhibits seed germination and seedling growth of canola more than neutral salt. Front. Plant Sci..

[7] Li J., Pu L., Han M., Zhu M., Zhang R., Xiang Y. (2014). Soil salinization research in China: Advances and prospects. J. Geogr. Sci..

[8] Parihar P., Singh S., Singh R., Singh V.P., Prasad S.M. (2015). Effect of salinity stress on plants and its tolerance strategies: A review. Environ. Sci. Pollut. Res..

[9] Hasanuzzaman M., Raihan M.R.H., Alharby H.F., Al-Zahrani H.S., Alsamadany H., Alghamdi K.M., Ahmed N., Nahar K. (2023). Foliar application of ascorbic acid and tocopherol in conferring salt tolerance in rapeseed by enhancing K^+^/Na^+^ homeostasis, osmoregulation, antioxidant defense, and glyoxalase system. Agronomy.

[10] Waadt R., Seller C.A., Hsu P.K., Takahashi Y., Munemasa S., Schroeder J.I. (2022). Plant hormone regulation of abiotic stress responses. Nat. Rev. Mol. Cell Biol..

[11] Koca H., Bor M., Özdemir F., Türkan İ. (2007). The effect of salt stress on lipid peroxidation, antioxidative enzymes and proline content of sesame cultivars. Environ. Exp. Bot..

[12] El-Badri A.M., Batool M., Mohamed I.A.A., Wang Z., Khatab A., Sherif A., Ahmad H., Khan M.N., Hassan H.M., Elrewainy I.M. (2021). Antioxidative and metabolic contribution to salinity stress responses in two rapeseed cultivars during the early seedling stage. Antioxidants.

[13] Zhang X., Long Y., Chen X., Zhang B., Xin Y., Li L., Cao S., Liu F., Wang Z., Huang H. (2021). A NAC transcription factor *OsNAC3* positively regulates ABA response and salt tolerance in rice. BMC Plant. Biol..

[14] Song W., Shao H., Zheng A., Zhao L., Xu Y. (2023). Advances in roles of salicylic acid in plant tolerance responses to biotic and abiotic stresses. Plants.

[15] Guo L., Yang H., Zhang X., Yang S. (2013). Lipid transfer protein 3 as a target of MYB96 mediates freezing and drought stress in arabidopsis. J. Exp. Bot..

[16] Yu L., Nie J., Cao C., Jin Y., Yan M., Wang F., Liu J., Xiao Y., Liang Y., Zhang W. (2010). Phosphatidic acid mediates salt stress response by regulation of MPK6 in *Arabidopsis thaliana*. New Phytol..

[17] Zhang W.-X., Liang X.-M., Dai C., Wen J., Yi B., Tu J.-X., Shen J.-X., Fu T.-D., Ma C.-Z. (2023). Genome editing of *BnaMPK6* gene by CRISPR/Cas9 for loss of salt tolerance in *Brassica napus* L. Acta Agron. Sin..

[18] Shi H., Zhu J.-K. (2002). Regulation of expression of the vacuolar Na^+^/H^+^ antiporter gene *AtNHX1* by salt stress and abscisic acid. Plant Mol. Biol..

[19] Mizoi J., Shinozaki K., Yamaguchi-Shinozaki K. (2012). AP2/ERF family transcription factors in plant abiotic stress responses. Biochim. Biophys. Acta.

[20] Shiroguchi K., Jia T.Z., Sims P.A., Xie X.S. (2012). Digital RNA sequencing minimizes sequence-dependent bias and amplification noise with optimized single-molecule barcodes. Proc. Natl. Acad. Sci. USA.

[21] Xiang T., Li J., Bao S., Xu Z., Wang L., Long F., He C. (2021). Digital RNA-seq transcriptome plus tissue anatomy analyses reveal the developmental mechanism of the calabash-shaped root in tetrastigma hemsleyanum. Tree Physiol..

[22] Luna Santamaría M., Andersson D., Parris T.Z., Helou K., Österlund T., Ståhlberg A. (2024). Digital RNA sequencing using unique molecular identifiers enables ultrasensitive RNA mutation analysis. Commun. Biol..

[23] Puga M.I., Mateos I., Charukesi R., Wang Z., Franco-Zorrilla J.M., De Lorenzo L., Irigoyen M.L., Masiero S., Bustos R., Rodríguez J. (2014). SPX1 is a phosphate-dependent inhibitor of phosphate starvation response 1 in arabidopsis. Proc. Natl. Acad. Sci. USA.

[24] Zhao L., Liu F., Xu W., Di C., Zhou S., Xue Y., Yu J., Su Z. (2009). Increased expression of *OsSPX1* enhances cold/subfreezing tolerance in tobacco and *Arabidopsis thaliana*. Plant Biotechnol. J..

[25] Jiangsu Provincial Bureau of Quality and Technical Supervision (2017). Technical Regulation for Identification and Evaluation of Salt Tolerance in Rapeseed (DB32/T 3278-2017).

[26] Bradford M.M. (1976). A rapid and sensitive method for the quantitation of microgram quantities of protein utilizing the principle of protein-dye binding. Anal. Biochem..

[27] Yu Z., Duan X., Luo L., Dai S., Ding Z., Xia G. (2020). How plant hormones mediate salt stress responses. Trends Plant Sci..

[28] Livak K.J., Schmittgen T.D. (2001). Analysis of relative gene expression data using real-time quantitative PCR and the 2^−ΔΔ*C_T_*^ method. Methods.

[29] Gietz R.D., Schiestl R.H. (2007). High-efficiency yeast transformation using the LiAc/SS carrier DNA/PEG method. Nat. Protoc..

[30] Cardoza V., Stewart C.N. (2024). Brassica biotechnology: Progress in cellular and molecular biology. Vitro Cell Dev. Biol. Plant.

[31] Chen C., Chen H., Zhang Y., Thomas H.R., Frank M.H., He Y., Xia R. (2020). TBtools: An integrative toolkit developed for interactive analyses of big biological data. Mol. Plant.

[32] Munns R., Tester M. (2008). Mechanisms of salinity tolerance. Annu. Rev. Plant Biol..

[33] Zhu J.-K. (2002). Salt and drought stress signal transduction in plants. Annu. Rev. Plant Biol..

[34] Raza A., Tabassum J., Fakhar A.Z., Sharif R., Chen H., Zhang C., Ju L., Fotopoulos V., Siddique K.H., Singh R.K. (2023). Smart reprograming of plants against salinity stress using modern biotechnological tools. Crit. Rev. Biotechnol..

[35] Oh M.-M., Trick H.N., Rajashekar C.B. (2009). Secondary metabolism and antioxidants are involved in environmental adaptation and stress tolerance in lettuce. J. Plant Physiol..

[36] Verma V., Ravindran P., Kumar P.P. (2016). Plant hormone-mediated regulation of stress responses. BMC Plant Biol..

[37] Agarwal P.K., Shukla P.S., Gupta K., Jha B. (2013). Bioengineering for salinity tolerance in plants: State of the art. Mol. Biotechnol..

[38] Zribi O.T., Barhoumi Z., Kouas S., Ghandour M., Slama I., Abdelly C. (2015). Insights into the physiological responses of the facultative halophyte aeluropus littoralis to the combined effects of salinity and phosphorus availability. J. Plant. Physiol..

[39] Dai R., Zhan N., Geng R., Xu K., Zhou X., Li L., Yan G., Zhou F., Cai G. (2024). Progress on salt tolerance in *Brassica napus*. Plants.

[40] Parida A.K., Das A.B. (2005). Salt tolerance and salinity effects on plants: A review. Ecotoxicol. Environ. Saf..

