# Supplementary figures and images for: Identification of Salt-Tolerant Germplasm and Salt-Responsive Genes in Brassica napus Through Phenotypic and Transcriptomic Analyses
Source: Antioxidants (Basel). 2026 Jul 22;15(7):909. doi: 10.3390/antiox15070909 (PMC13406129; doi:10.3390/antiox15070909)

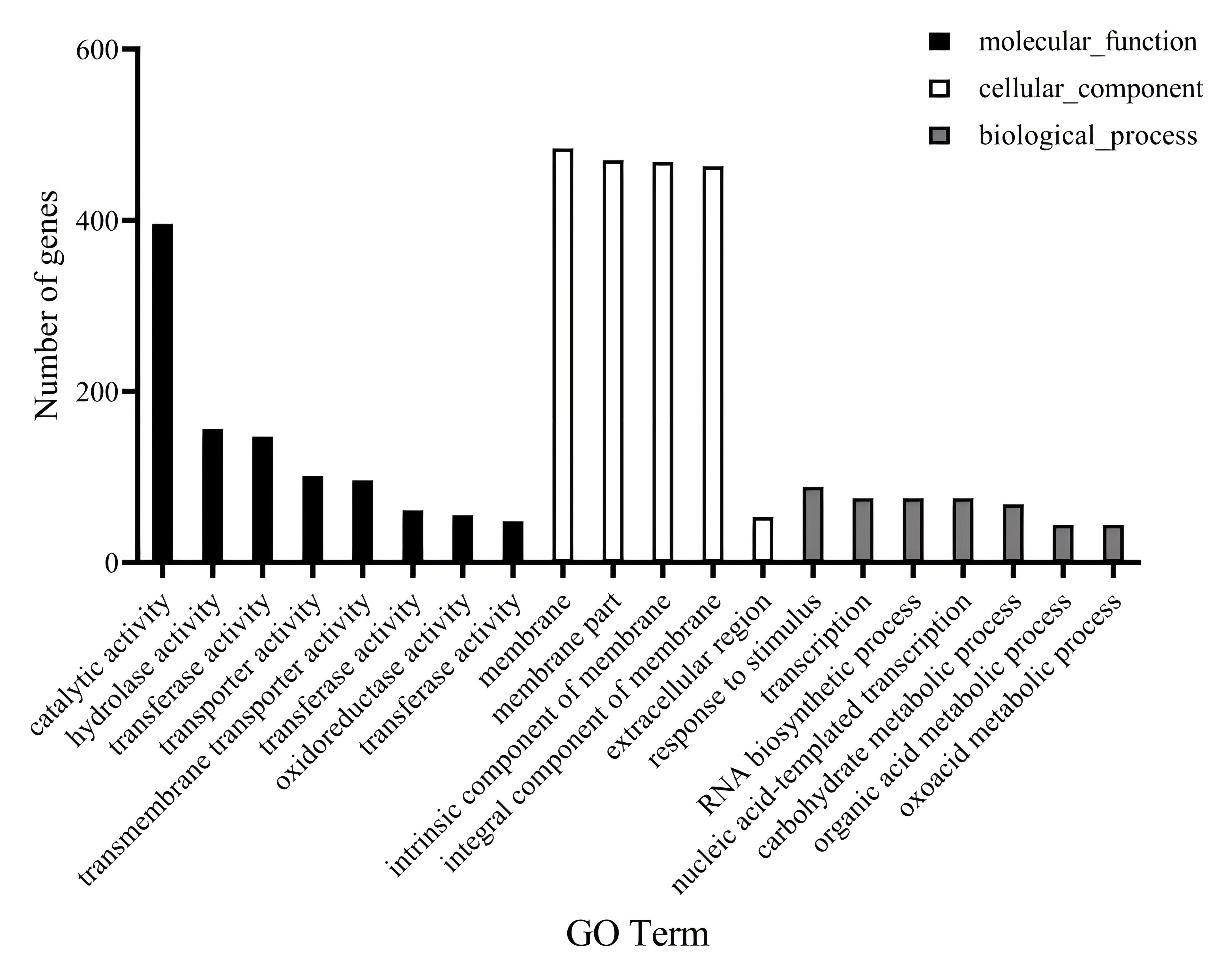

Supplement: Supplementary file 1 [file antioxidants-15-00909-s001.zip › antioxidants-4417741-supplementary/Supplementary Figure S1A GO enrichment of DEGs under Y1.png]

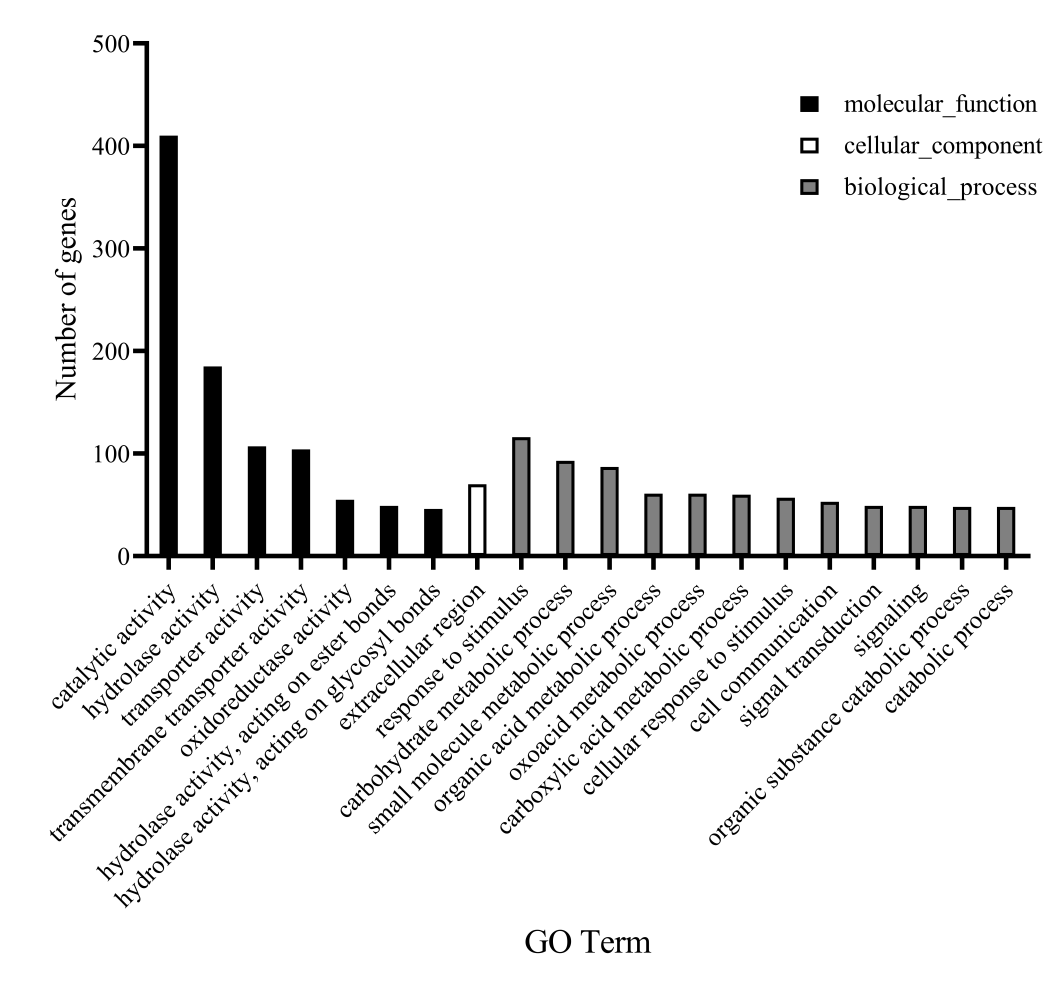

Supplement: Supplementary file 1 [file antioxidants-15-00909-s001.zip › antioxidants-4417741-supplementary/Supplementary Figure S1B GO enrichment of DEGs under Y2.png]

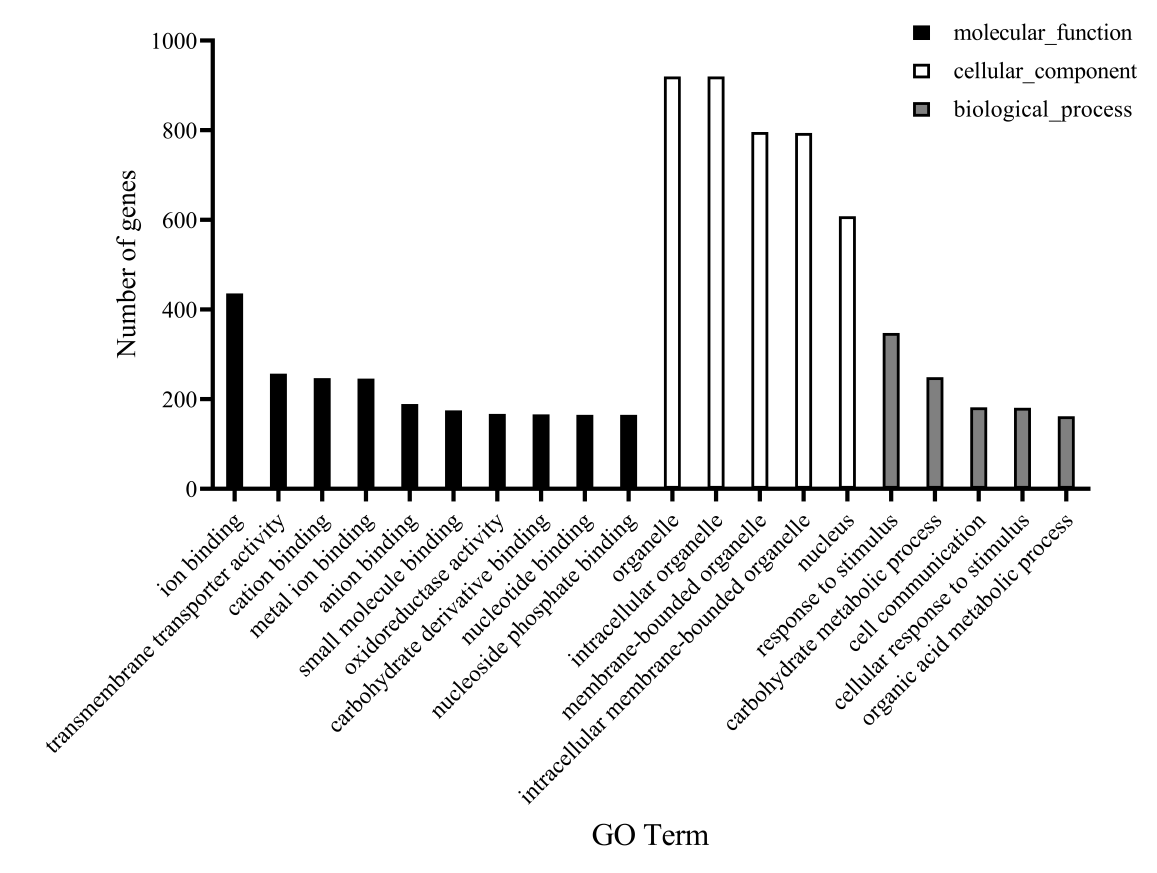

Supplement: Supplementary file 1 [file antioxidants-15-00909-s001.zip › antioxidants-4417741-supplementary/Supplementary Figure S1C GO enrichment of DEGs under Y3.png]
